# The Link between Oxidative Stress, Redox Status, Bioenergetics and Mitochondria in the Pathophysiology of ALS

**DOI:** 10.3390/ijms22126352

**Published:** 2021-06-14

**Authors:** Elena Obrador, Rosario Salvador-Palmer, Rafael López-Blanch, Ali Jihad-Jebbar, Soraya L. Vallés, José M. Estrela

**Affiliations:** Department of Physiology, Faculty of Medicine and Odontology, University of Valencia, 15 Av. Blasco Ibañez, 46010 Valencia, Spain; rosario.salvador@uv.es (R.S.-P.); loblanch@alumni.uv.es (R.L.-B.); aji.jebbar@gmail.com (A.J.-J.); lilian.valles@uv.es (S.L.V.)

**Keywords:** ALS, mitochondria, bioenergetics, redox status, oxidative stress

## Abstract

Amyotrophic lateral sclerosis (ALS) is the most common neurodegenerative disease of the motor system. It is characterized by the degeneration of both upper and lower motor neurons, which leads to muscle weakness and paralysis. ALS is incurable and has a bleak prognosis, with median survival of 3–5 years after the initial symptomatology. In ALS, motor neurons gradually degenerate and die. Many features of mitochondrial dysfunction are manifested in neurodegenerative diseases, including ALS. Mitochondria have shown to be an early target in ALS pathophysiology and contribute to disease progression. Disruption of their axonal transport, excessive generation of reactive oxygen species, disruption of the mitochondrial structure, dynamics, mitophagy, energy production, calcium buffering and apoptotic triggering have all been directly involved in disease pathogenesis and extensively reported in ALS patients and animal model systems. Alterations in energy production by motor neurons, which severely limit their survival capacity, are tightly linked to the redox status and mitochondria. The present review focuses on this link. Placing oxidative stress as a main pathophysiological mechanism, the molecular interactions and metabolic flows involved are analyzed. This leads to discussing potential therapeutic approaches targeting mitochondrial biology to slow disease progression.

## 1. Introduction

ALS is a rapidly progressive neurodegenerative disease [1] characterized by a gradual loss of upper and lower motor neurons (MNs). The consequence of the loss of MNs in the cerebral cortex, brainstem and medulla is a muscular weakness that progresses, threatening motor autonomy, oral communication, swallowing and/or breathing. The disease does not affect eye muscles, sphincter control or skin sensitivity, nor does it usually cause damage to the patient’s cognitive ability, although approximately 10–15% of cases may present signs of frontotemporal dementia, and in more than 35% of cases, signs of cognitive impairment are detected [2].

The most common form of ALS is called sporadic ALS (SALS) and can affect anyone regardless of gender or ethnicity, although it most often affects people between the ages of 40 and 70. The other type of ALS (5 to 10%) is called familial ALS (FALS), when there is a previously diagnosed first- or second-degree family history [1]. The onset will be spinal in the form of distal weakness in the hands or lower limbs. The slow initial progression and the absence of pain cause patient delay in seeking medical advice. The appearance of abnormal muscle movements (spasms, jerks, cramps or weakness) in the hands and feet causes difficulty in lifting, walking or using one’s hands to dress, wash and button clothing. It can also start with an abnormal loss of muscle mass or body weight [2,3]. The progression of the disease is normally asymmetric. Sometimes, it is very slow, developing over the years and having periods of stability with a variable degree of disability. Other times, its development is rapid [3]. More striking is the bulbar onset, with impaired speech or swallowing. Approximately 3% of cases will make a respiratory debut. Gordon et al. [4] also proposed a diagnostic classification of upper MN-predominant MN disease that mainly distinguishes between clinically pure primary lateral sclerosis and a new category named upper MN-dominant ALS, where patients show spastic paralysis, hyperreflexia, minor electromyogram denervation or lower MN signs on examination and a slower disease progression compared to those with typical ALS [4]. Therefore, ALS should be considered a clinical syndrome, with different forms of presentation and, likely, with multiple risk factors. Regardless of the type of presentation, the ALS patient becomes totally dependent until death, generally due to respiratory failure [5].

Since the vast majority of patients do not have the inherited type of ALS, the diagnosis of ALS is not determined by genetic testing. Unfortunately, there is no specific test that provides a diagnosis, meaning it is currently based on clinical symptoms, the presence in the physical examination of signs of first and second MNs and the electromyographic examination according to criteria from the El Escorial World Federation of Neurology (http://wfnals.org accessed on 5 June 2021). The rest of the clinical examination and tests will be conducted to exclude diseases that can present similar symptoms and are potentially treatable [6].

The majority of patients do not have a family history of ALS [7,8]. Although more than 100 potentially causative or disease-modifying genes have been identified, the most common mutations are found in C9orf72 (chromosome 9 open reading frame 72), SOD1 (superoxide dismutase 1), FUS (fused in sarcoma/translocated in liposarcoma or heterogeneous nuclear ribonucleoprotein P2) and TARDBP (transactive response DNA binding protein 43) (60–70% of all FALS cases) [9]. Inheritance, in most cases, is autosomal dominant, although autosomal recessive and X-linked dominant familial ALS can also occur [10]. Nevertheless, not all family members with the same mutations necessarily develop FALS. On the other hand, in the dark well of possible risk factors (causes) of SALS, there is a large list of suggested factors, i.e., (but not limited to) neurotoxins, chemicals, metals, radiation and electromagnetic fields, sport-related stress, non-equilibrated dietary habits, infectious agents, smoking, autoimmunity or traumatic brain injuries [11]. Most or all of them could be just contributing or enhancing factors in the complex pathophysiology that leads to the death of MNs. Some of the pathological alterations could even begin before the onset of motor symptoms. Whatever the real course of the pathophysiology, many of the abovementioned factors directly or indirectly converge in the mitochondria.

## 2. Mitochondrial Dysfunction at the Core of ALS Pathophysiology

Mitochondria have multiple functions that are essential for MN survival. Alterations in the structure and function of mitochondria have been postulated as the core in the pathophysiology of different neurodegenerative diseases [12] and are also evident in MNs affected by ALS [13,14,15,16]. This postulate is primarily based on disease models. However, postmortem studies also reported mitochondrial alterations in the medulla of patients with ALS, including (but not limited to) an abnormal cellular distribution (most mitochondria are found in the soma or proximal axon of MNs) [17], swollen and vacuolated mitochondria, a decrease in mitochondrial DNA [18] or a decrease in the activity of electron chain complexes and the activity of some mitochondrial enzymes [19]. Dysfunction of the electron transport chain may result in altered mitochondrial O_2_ consumption, increased generation of reactive oxygen species (ROS), which is associated with oxidative-related damage including changes in protein carbonylates and tyrosine nitration, decreased ATP synthesis and impaired DNA repair [20]. For instance, glutamate receptor-mediated neurotoxicity has been linked to an overload of mitochondrial Ca^2+^ and ROS production in cultured spinal motor neurons from transgenic ALS animals [21]. Excessive (dynamin-related protein 1 (Drp1) hyperactivation-associated) mitochondrial fission, fragmentation and dysfunction were reported in patient-derived fibroblasts and cultured motor neurons of several familial forms of ALS expressing mutant SOD1 [22]. MNs expressing mutant SOD1 show higher DNA damage compared with those expressing wild-type SOD1, possibly because of a loss of nuclear protection. The toxicity of mutant SOD1 might, hypothetically, arise from an initial misfolding reducing nuclear protection from the active enzyme. Importantly, in both cell models, inhibition of Drp1/Fis1 (mitochondrial fission 1 protein) interaction by a selective peptide inhibitor, P110, led to a significant reduction in ROS levels, and to improvement in mitochondrial structure and functions [22]. While ROS can increase Drp1, leading to sustained mitochondrial fission and fragmentation, a decrease in ATP levels can impair autophagy and proteasomal degradation. This can worsen protein aggregates and trigger endoplasmic reticulum (ER) stress [23]. All these alterations are presumably interconnected and, thus, form part of a cascade of molecular events leading to MN degeneration and death.

Nevertheless, in vivo data from patients were lacking until a recent study, using ^31^P-magnetic resonance spectroscopy, showed mitochondrial dysfunction in the brains and muscles of individuals living with ALS [24]. As an example, cohort studies report a significantly higher number of cases of ALS in professional soccer and American football players, as well as a slightly increased risk of ALS in varsity athletes [25]. Moreover, participation in an extra 10 kJ/kg/day of physical activity—equivalent to approximately 45 min brisk walking—was consistently associated with an increased risk of ALS, with the strongest association observed for adulthood exercise-related physical activity [26]. These facts suggest that a high-activity lifestyle could be associated with an elevated risk of ALS. In this regard, mitochondrial dysfunction could be the common factor linking over-trained athletes and patients with neurodegenerative diseases [27]. This suggestion could also lead to hypothesizing a correlation between mitochondrial dysfunction and excessive stress [28]. However, a recent study showed that people with ALS reported no raised levels of potentially stressful premorbid life events or occupational stress and did not have reduced levels of resilience [29]. Therefore, a possible role of exceptional physical or psychological stress (at least by itself) in the pathogenesis of ALS is still an open question. Without ruling out stress as a potentiating factor, the necessary cooperation of one or more other factors seems a plausible option. It is also a particularly intriguing question as to what mechanism may link stress to mitochondria. 

Although it is generally accepted that mitochondrial dysfunction is an early alteration in ALS [14], it is still unclear whether that dysfunction occurs at the beginning or is just the consequence of a cascade of pathological events that ultimately cause degeneration and death of MNs. Apoptotic cell death is linked to mitochondrial dysfunction and involves the release of mitochondrial death signals (intrinsic pathway) or may occur through an extrinsic pathway initiated by the binding of specific ligands to plasma membrane cell tumor necrosis factor (TNF)-related (neuroinflammation-related) death receptors such as FAS (apoptosis antigen 1) and death receptor 6 (DR6), thereby triggering activation of caspase-8 which in turn activates other executioner caspases [30]. Caspase 8-mediated cleavage of the Bid (BH3 interacting-domain death agonist) protein can also cause the release of mitochondrial proapoptotic proteins [31]. Additionally, as the consequence of an eventual energy/metabolic catastrophe, MN death can also be necrotic [32]. However, progression of the disease associates with other types of MN death, which may even be predominant. Apoptotic MN death has been detected postmortem in the medulla of ALS patients [33]. For instance, compared to healthy individuals, increased levels of protein p53 in MNs of the affected regions of ALS patients have been found [34], and this associates with a decrease in Bcl2 (B-cell lymphoma 2 protein) and an increase in the Bcl2-related Bax, Fas and caspases-8 and -3 proteins [35]. Increased levels of p53 in ALS patients suggest a possible down-regulation of proteins that negatively regulate p53 (e.g., murine doble minute 2 (mdm2)) or a reduced degradation of p53 by the proteasome (a mechanism damaged in neurodegenerative diseases and, in part, responsible for the accumulation of abnormal protein aggregates) [36]. Furthermore, in a mouse model of FALS, neuronal deletion of proapoptotic Bax and Bak slowed down neuronal loss, axonal degeneration, symptom onset, weight loss and paralysis and extended survival [37]. These examples suggest that damaged MNs activate the mitochondrial apoptotic pathway early in the disease process. In addition, other forms of cell death, i.e., necroptosis and pyroptosis, have also been implicated in ALS. Mechanisms that are directly related to oxidative stress and the inflammation-related cytokine storm associated with the pathophysiology of progressive ALS. For instance, ALS-derived astrocytes secrete neurotoxic factors that selectively kill MNs in a Bax-dependent mechanism [38], whereas TDP-43-mediated activation of microglia was shown to cause an NF-κB (nuclear factor kappa-light-chain-enhancer of activated B cells), AP-1 (activator protein 1) and NLRP3 (NLR family pyrin domain containing 3) inflammasome-dependent pro-inflammatory cascade that was toxic to MNs [39]. It is particularly noteworthy that in the absence of microglia, TDP-43 was not toxic to MNs [39].

Based on the many facts suggesting mitochondrial dysfunction as a key phenomenon in the pathophysiology of ALS, the aim of this discussion is to present evidence linking oxidative stress to alterations in the redox status, bioenergetics and mitochondrial physiology, and the consequent MN death.

## 3. Glia, Neuroinflammation and Motor Neuron Mitochondria

Neuroinflammation has been consistently observed in motor regions of the central nervous system (CNS) in SALS and FALS, constituting a hallmark of the disease [40]. 

Astrocyte and microglial dysfunctions have been demonstrated in patients and animal models of ALS [41]. Normal astrocytes promote a physiological environment for neuronal function. However, astrocytes in ALS have the potential to be harmful to MNs. In ALS patients and animal models, astrocytes become enlarged and abnormally proliferate towards reactive astrogliosis that surrounds the degenerating MNs [42]. This can act like a scar and produce inhibitory molecules that block the regrowth of damaged axons [43]. Markers of axonal damage (tau protein, heavy-chain neurofilaments (NfHSMI35) and progranulin) are increased in the cerebrospinal fluid of ALS patients [44,45]. The presence of axonal spheroids and perikaryal accumulations/aggregations (comprising the neuronal intermediate filament proteins) have been observed in sporadic cases of ALS as well as in mutant SOD1 patients, likely interfering with axonal transport [46]. Mitochondrial dysfunction occurs at an early stage of axon degeneration [47]. ALS-associated mitochondrial bioenergetic deficits are evident in the most common ALS-causing mutation, C9orf72 [48]. Interestingly, C9orf72 MNs also show shorter axons and impaired fast axonal transport of mitochondrial cargo [48]. Importantly, it has been shown that PGC1α (peroxisome proliferator-activated receptor gamma coactivator 1-alpha) overexpression improves mitochondrial metabolism, and that appears enough to restore axonal dynamics [48]. Since PGC1α regulates the genes involved in energy metabolism [49] and is also a master regulator of mitochondrial biogenesis [50], this transcriptional co-activator seems to establish a clear link between mitochondrial bioenergetics and axonal transport. Moreover, PGC-1α regulates the expression of mitochondrial antioxidant genes, including SOD2 (superoxide dismutase 2), catalase, peroxiredoxins 3 and 5, uncoupling protein 2, thioredoxin 2 and thioredoxin reductase, and, thus, is involved in preventing oxidative injury and mitochondrial dysfunction [51].

Microglia cells represent the first and primary form of active immune defense in the CNS and can be activated by any type of toxic challenge, including injury, infection and ischemia [52]. Upon activation, they enlarge, migrate and can become phagocytic to remove toxic matter. Activated microglial cells secrete pro-inflammatory and neurotoxic factors [53]. If the microglia cells cannot resolve or eliminate a toxic insult in the CNS, they remain reactive and continue to recruit astrocytes and oligodendrocytes, causing an ongoing inflammatory process [54]. In rodents expressing the ALS-linked SOD1^G93A^ mutation (a G93A transgene mutant form of human SOD1), rapid spread of paralysis coincides with the emergence of neurotoxic and proliferating aberrant glia cells with an astrocyte-like phenotype (found surrounding the damaged MNs) [55]. However, selective NF-κB inhibition in ALS astrocytes was not found sufficient to rescue motor neuron death [56]. However, the deletion of NF-κB signaling in microglia rescued MNs from microglial-mediated death in vitro and extended survival in ALS mice [56], thus suggesting anti-inflammation and inhibition of microglia-induced toxicity as a potential therapeutic strategy. 

Furthermore, in rat astroglial cultures exposed to the cerebrospinal fluid from ALS patients, enhanced production and release of inflammatory cytokines IL6 (interleukin 6) and TNFα, as well as increased production of cyclooxygenase-2 (COX-2) and prostaglandin E2 (PGE2), have been observed. Concomitantly, anti-inflammatory cytokine IL10 and the beneficial trophic factors VEGF (vascular endothelial growth factor) and GDNF (glial cell-derived neurotrophic factor) were down-regulated [57]. Moreover, in experiments where reactive/aberrant astrocytes were co-cultured with MNs, neuron survival was less than 10% [58], thus suggesting a non-permissive microenvironment for neuron growth and differentiation. Human astrocytes derived from the spinal cord of persons with SALS or FALS also killed MNs in culture [59].

There is evidence linking mitochondrial dysfunction and neuroinflammation [16,60,61]. Different studies have found a link between TANK-binding kinase 1 (TBK1) mutations and ALS. TBK1 is an inducer of type-1 interferons and also has a key role in autophagy and mitophagy [62]. Fragmented mitochondria also fuel neuroinflammation [63]. For instance, mitochondria fission was found to be mediated by the Drp1-Fis1 signaling pathway, which was blocked by P110 [63], thus suggesting that targeted inhibition of mitochondria fission may represent a new strategy for preventing nerve cell death in ALS. Moreover, in a recent contribution where the effect of an NAD^+^ booster (nicotinamide riboside) and a natural antioxidant (pterostilbene) was assayed in ALS SOD1^G93A^ mice, the interdependence among neuroinflammation, mitochondria and the activation of apoptotic cell death in MNs was clearly described [64]. Importantly, nicotinamide riboside and pterostilbene also decreased the microgliosis and astrogliosis associated with ALS progression [64].

## 4. Oxidative Stress, Redox Status, Bioenergetics and Mitochondria in the Mechanisms Leading to the Death of Motor Neurons

### 4.1. Oxidative Stress, Redox Status and Mitochondria

Oxidative stress, capable of damaging all components of a cell, is the consequence of an increased generation of ROS and/or a decrease in the effectiveness of antioxidant defenses [65]. Although ROS may not be the triggering factor for neurodegenerative diseases (including ALS), they are likely to exacerbate disease progression [40]. Resident glial cells and infiltrated immune cells are considered among the major producers of ROS and reactive nitrogen species (RNS) in pathological conditions of the CNS, including MN diseases [66]. NO and H_2_O_2_ produced in excess can lead to the formation of potent oxidants, such as .OH and ^-^OONO radicals, via a trace metal-dependent process [67]. 

Besides MNs, mitochondria, as it occurs in any cell, are the primary site of energy generation, but also the main source of ROS. Under conditions in which mitochondrial generation of ROS is increased (such as in the presence of Ca^2+^ ions or when the mitochondrial antioxidant defense mechanisms are compromised), these reactive species may lead to irreversible damage of mitochondrial DNA, membrane lipids and proteins. Oxidative stress-induced damage can impair mitochondrial function and ultimately cause cell death [68]. 

Taken as an example, the SOD1 mutations do not cause a loss of function when the protein is fully loaded with Cu^2+^ or Zn^2+^. The SOD1 mutation does have a lower affinity for Zn^2+^ [69], and an altered Cu^2+^ co-ordination makes Zn-deficient SOD1 a more efficient oxidant, since it donates an electron to O_2_ to generate O_2_.^-^ at the Cu^2+^ catalytic site which then reacts with NO to form peroxynitrite (very damaging to the CNS) [70]. Interestingly, increased activities of SOD1 and glutathione peroxidase have been observed in neurons expressing higher levels of cellular PrP^C^ (a Cu^2+^-binding prion protein, which can incorporate varying amounts of Cu^2+^ and exhibit protective antioxidant activity [71]), and a role of PrP^C^ in the cellular defense against oxidative stress (or other cellular stresses) has been proposed [72,73,74]. Therefore, conformational changes of the normal (cellular) form of PrP^C^ to a disease-associated form could affect MN antioxidant defense. 

In transmissible spongiform encephalopathies, the infectious agent or “prion” is thought to be PrP^res^ [75]. PrP^res^ is a protease-resistant conformer of PrP^C^. Yuan et al. [76] reported that normal PrP^C^ transformed into a protease K-resistant protein under oxidative stress in the presence of the human prion neuropeptide PrP106–126. Mitochondrial damage and dysfunction in prion disease progression were also observed in this study, thus implicating this damage as causal for a given prion disease (as ALS could hypothetically be).

The redox state is often used to describe the balance of glutathione disulfide/glutathione (GSSG/GSH), NAD^+^/NADH and NADP^+^/NADPH in cells and is reflected in the balance of several sets of metabolites (e.g., lactate and pyruvate, β-hydroxybutyrate and acetoacetate), whose interconversion is dependent on these ratios (e.g., Jones 2015 [77]). For instance, GSH levels (the main non-protein thiol and a prevalent antioxidant in mammalian cells) are lower in the motor cortex of ALS patients as compared to healthy volunteers [78]. It has been shown that GSH depletion promotes neurological deficit, mitochondrial pathology [79] and MN degeneration [80] in mutant SOD1 ALS mice. Regarding toxic protein aggregates, Meyerowitz et al. observed that chronic oxidative and nitrosative stress induced several features consistent with TDP-43 (the polypeptide product of the TARDBP gene) proteinopathies including loss of nuclear TDP-43, accumulation of diffuse TDP-43 in the cytosol, formation of a 35-kDa C-terminal fragment and accumulation of TDP-43 in RNA stress granules, some of which revealed ubiquitination [81]. Both sporadic and familial forms of ALS have the pathologic TDP-43 signature of abnormal hyperphosphorylation, ubiquitination and C-terminal fragments in affected brains and spinal cords, suggesting that they share a common mechanism of pathogenesis. Importantly, it has also been observed that TDP-43 deposition leads to targeted RNA instability in ALS and may ultimately cause cell death by disrupting energy production and protein synthesis pathways [82].

Using TDP-43 as an example, it is interesting that wild-type and mutant TDP-43 aggregation is caused by incorrect disulfide bonds involving Cys residues in one of its RNA recognition motifs, and aggregation is induced by oxidative stress [83,84]. TDP-43 clumps have been found in 90% of patients with SALS, and in ALS patients with mutations in several ALS-associated genes, including TARDBP and PFN1 (profilin 1). Interestingly, these aggregates are rarely seen in patients with mutations in the SOD1 gene, which account for 12–20% of FALS cases. Mitoautophagy (a self-destructive path involving mitochondria’s elongation and the formation of a ring-like structure before its disintegration) was found in upper MNs of ALS mice, particularly those with TARDBP or PFN1 mutations, supporting an association between ALS with TDP-43 toxic aggregates and mitochondrial dysfunction [85]. All these pathological mechanisms suggest a clear link between oxidative stress, abnormal protein aggregation and the accumulation within the MNs of damaged mitochondria. In fact, MNs from mice expressing wild-type human TDP-43 have cytoplasmic TDP-43-positive inclusions composed of mitochondria aggregates [86]. In agreement with this experimental evidence, it has been shown that inhibition of TDP-43 mitochondrial localization blocks its neuronal toxicity [87], and that TDP-43 aggregation induced by oxidative stress causes a global mitochondrial imbalance in ALS [88]. 

Furthermore, formation of abnormal neuronal mitochondrial cristae was found in heterozygous TDP-43 (A315TKi) mutant animals [89]. TDP-43 also perturbs ER–mitochondria interactions, and this is associated with disruption in cellular Ca^2+^ homeostasis [90]. This disruption may have particular relevance since it is well known that key functions of mitochondria (matrix dehydrogenases, in particular) and morphology are strongly affected by an increase in Ca^2+^ within the matrix [91,92]. Moreover, expression of mutant TDP-43 in an MN-like cell line induces oxidative stress, mitochondrial damage and nuclear accumulation of nuclear factor E2-related factor 2 (Nrf2, a key regulator of the antioxidant defense and other protective mechanisms) [93]. As pointed out by Mimoto et al. [94], Nrf2 dramatically increased by 2-fold in the nucleus of MNs at 14 weeks (early symptomatic) and by almost 5-fold at 18 weeks (end symptomatic) in Tg mice. Additionally, studies in ALS mouse models have shown a significant beneficial effect of elevated Nrf2 levels in astrocytes, the main GSH suppliers for neurons [95]. Furthermore, treatment with Nrf2 activators, e.g., cyanoenone triterpenoids, decreases neuroinflammation in mouse models of ALS [96], thus suggesting that Nrf2 signaling is also critical to attenuating neuroinflammation in ALS through repression of the deleterious effects of activated glial cells on neurons.

Mitochondrial alteration can also be caused by other ALS-related proteins. For example, mutations in valosin-containing protein (VCP, a member of the type II AAA+ ATPase family with a number of cellular functions including mitochondrial quality control, autophagy, vesicle transport and fusion, 26S proteasome function and assembly of peroxisomes) are found in 2% of all FALS patients [97]. It has also been reported that VCP deficiency causes significant mitochondrial uncoupling, leading to decreased mitochondrial membrane potential, increased mitochondrial oxygen consumption and reduction in cellular ATP production [98]. 

Overexpression of ALS-linked human mutant FUS also leads to Golgi fragmentation and mitochondria aggregation in rats [99]. FUS also interacts with PGC-1α, which is down-regulated in the SOD1^G93A^ mouse model and in SALS patients [100]. Moreover, a decrease in PGC-1α associates with a lower expression of sirtuin (Sirt) 3 [101], a main mitochondrial deacetylase. Overexpression of Sirt3 in cultured cells increases respiration and expression of PGC-1α and decreases the production of ROS [101,102]. Sirt3 and PGC-1α also show protective effects against mitochondrial fragmentation in spinal cord MNs [101]. 

**Figure 1:**
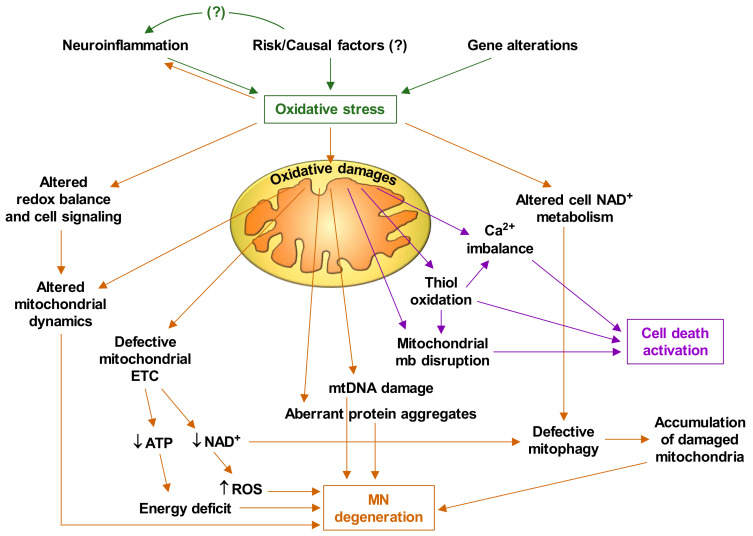
Oxidative stress-induced motor neuron degeneration and death. ALS is caused by interplay of various molecular pathways in motor neurons and an interaction with neighboring glial cells and different undefined risk/causal factors. ETC, electron transport chain; mtDNA, mitochondrial DNA; mb, membrane. It is suspected that this cascade of events gradually damages motor neurons until (a still undefined) a-point-of-no-return threshold is reached and cell deterioration and death are irreversible [14].

Based on the experimental facts discussed thus far, and the abundant available literature on mitochondrial alterations in ALS, it is feasible to deduce that oxidative stress plays a central role in the pathophysiology of ALS. Figure 1 briefly describes how oxidative stress triggers mitochondrial dysfunctions and the cascade of events that leads to MN degeneration and death. 

### 4.2. Bioenergetics and Mitochondria 

ALS is frequently associated with several defects in energy metabolism, including weight loss, hypermetabolism and hyperlipidemia [103]. Several epidemiological studies have identified diets that positively affect ALS patients, including various high-calorie fat or sugar-based diets [104]. However, a substantial proportion of patients with ALS develop glucose intolerance [105,106]. Different preclinical studies also show problems affecting glucose transport and metabolism (recently reviewed by Tefera et al. [107]), which imply progressive reductions in the capacity of MNs to generate energy from glucose, a metabolic problem that does not seem to affect, e.g., neighboring astrocytes, as shown in the SOD1^G93A^ ALS model [108]. In the event that the obtaining of energy from glucose progressively fails, it is obvious that MNs need an alternative source of energy to survive. At present, the only proven alternative are ketone bodies. Based on this assumption, results obtained in the SOD1^G93A^ ALS model suggest that a ketogenic diet may delay the progression of the clinical and biological manifestations of ALS [109]. Since ketone bodies can exert neuroprotective effects, the moderate ketosis induced by regular medium-chain fatty acid ingestion may have neuroprotective potential [109,110]. Interestingly, a high intake of polyunsaturated fatty acids and vitamin E appears associated with a 50–60% decreased risk of developing ALS, and these nutrients appear to act synergistically [111]. Moreover, in a follow-up of 995 ALS patients documented, a greater ω-3 polyunsaturated fatty acid intake was also associated with a reduced risk of ALS [112]. Importantly, β-hydroxybutyrate has been shown to inhibit mitochondrial ROS production in stressed neurons by facilitating NADH oxidation [113]. NADH oxidation and, consequently, the increased NAD^+^/NADH ratio have important implications in cellular redox homeostasis and the activation of protein deacetylases such as Sirt1 and Sirt3 [114]. A new clinical trial, Efficacy and Tolerability of Beta Hydroxybutyrate in Patients With Amyotrophic Lateral Sclerosis (ALS) (KETO-ALS) (NCT04820478, www.clinicaltrials.gov accessed on 5 June 2021), is now open for patient recruitment. This trial has been planned based on a previous one providing preliminary evidence that high-caloric nutrition might prolong survival in fast-progressing ALS patients (LIPCAL-ALS, (NCT02306590)). The KETO-ALS study suggests that a ketogenic diet is difficult to implement in ALS as it requires a long-term change in eating habits, which is difficult to achieve due to progressive dysphagia, fast worsening of patients’ general condition and limited survival. Therefore, the direct administration of ketone bodies may represent a more realistic alternative in ALS as it is easy to apply and allows maintaining patients’ usual eating habits.

Sirt3 has been shown to regulate ketone body production by deacetylating mitochondrial 3-hydroxy-3-methylglutaryl CoA synthase 2 (HMGCS2) [115], a fact in agreement with the elevation of mitochondrial Sirt3 expression in the spinal cord of SOD1^G93A^ mice following medium-chain triglyceride treatment [116], thus suggesting that medium-chain triglycerides may influence mitochondrial activity and survival of MNs through a Sirt3-dependent mechanism. In addition, Sirt1 has also been shown to de-acetylate and affect the activity of both members of the PGC-1α/ERR-α (estrogen-related receptor alpha) complex [117], which regulate mitochondrial biogenesis, and genes involved in gluconeogenesis, oxidative phosphorylation and fatty acid metabolism [118,119]. 

On the other hand, some antioxidants have shown potential beneficial effects in animal models; however, human clinical trials of antioxidant therapies have, thus far, been disappointing [120]. Nevertheless, in a recent pilot study, we demonstrated that the association of pterostilbene (PT, a natural antioxidant polyphenol) and nicotinamide riboside (NR, a vitamin B3 derivative and an NAD^+^ booster that supports the activity of Sirts) was able to slow the progressive decline in functionality, strength and lung function in ALS patients [121]. Importantly, we recently observed in MNs isolated from mutant SOD1^G93A^ mice that the NR- and PT-induced decrease in the release of proapoptotic signals associates with increased (autophagosome-bound) LC3-II (microtubule-associated proteins 1A/1B light chain 3B) along with decreased SQSTM1/P62 (sequestosome 1) levels, a strong indication that autophagy is induced [57]. These results also associated with an NR- and PT-induced increase in the percentage of lysosomes that colocalized with mitochondria in the MNs, which clearly suggests an increased mitophagy [57]. Indeed, defective mitophagy leads to the accumulation of damaged mitochondria and cellular dysfunction, thus favoring neurodegeneration [122]. This is important because the ALS pathophysiology may cause the accumulation of damaged mitochondria and, consequently, an energetic failure (Figure 1). The pilot study mentioned above [100] has been complemented by data obtained in patients treated with PT, NR and coconut oil, which is rich in medium-chain fatty acids (which are absorbed directly into the blood via intestine capillaries). Importantly, patients treated with the triple combination (PCT/US18/32932) and followed up for a period of 4 years show the highest survival rates (Estrela JM et al., unpublished data).

**Figure 2:**
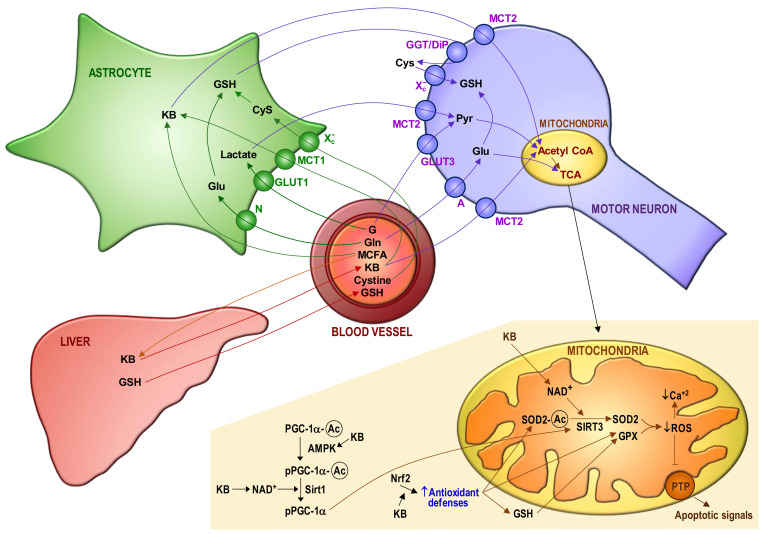
Blood–astrocyte–motor neuron metabolite fluxes and the redox-dependent control of apoptosis activation in motor neurons. The main metabolite fluxes are displayed. A progressive decrease in the capacity of motor neurons (MNs) to use glucose (G) may be complemented by ketone bodies (KB) as a source of energy. Plasma glutathione (GSH) (the liver is its major reservoir) and the GSH synthesized and released by astrocytes are the main sources of GSH for MNs. The control of mitochondrial reactive oxygen species (ROS) mainly depends on Mn superoxide dismutase (SOD2), GSH peroxidase (GPX) and mitochondrial GSH (which is not synthesized within the organelle and must be imported from the cytosol through a multicomponent transport system). GSH levels in mitochondria are also maintained by GSH reductase and NADPH, an essential reducing equivalent for enzyme-linked GSH recycling [123]. Moreover, the NADPH-dependent thioredoxin reductase/thioredoxin–peroxyredoxin/sulfiredoxin coupled systems also contribute to control ROS and cellular thiol homeostasis [124,125]. Mitochondrial sources of NADPH are the nicotinamide nucleotide transhydrogenase, isocitrate dehydrogenase-2 and malic enzyme. The mitochondrial GSH transport system suffers from progressive damage [64], presumably due an excess in ROS production and the inhibition exerted by the cytosolic glutamate (Glu). We observed that cytosolic Glu levels are increased in MNs isolated from mutant FUS R521C mice, as compared to the levels measured in MNs isolated from control wild-type mice [16] (which suggests an increase in glutamine (Gln) uptake and metabolism). The increase in cytosolic Glu is enough to partially inhibit the transport of GSH from the cytosol to mitochondria. Mitochondrial GSH depletion and Ca^2+^ load may initiate the cascade of events leading to MN death, including mitochondrial dysfunction, oxidative/nitrosative stress-associated damage, formation of Ca^2+^-rich precipitates and the release of proapoptotic molecules to the cytosol. Different molecular mechanisms control the antioxidant defense of MNs and the generation of ROS by mitochondria, i.e., PGC-1 α (peroxisome proliferator-activated receptor γ co-activator 1 α), Nrf2 (nuclear factor erythroid 2-related factor 2) and sirtuins (Sirt) 1 and 3. Sirt3 can be post-translationally modified through lipoperoxide-induced carbonylation, which results in loss of activity [126]. The steps where NAD^+^ and KB may interact to prevent apoptosis activation are indicated. AMP-activated protein kinase, AMPK; medium-chain fatty acids, MCFA; amino acid transporter system N, N; amino acid transporter system A, A; glucose transporter, Glut; monocarboxylate transporter, MCT; cysteine/glutamate transporter, Xc^-^; γ-glutamyl transpeptidase/dipeptidase, GGT/DIP; lactate, Lact; pyruvate, Pyr; tricarboxylic acid cycle, TCA; permeability transition pore complex, PTP; acetylated, Ac.

Figure 2 schematizes potential interrelationships between mitochondria and bioenergetics in MNs of ALS patients. The consequences of mitochondrial dysfunction (collapse of the mitochondrial inner transmembrane potential, uncoupling of the respiratory chain, hyperproduction of ROS, disruption of mitochondrial biogenesis, outflow of matrix Ca^2+^, mitochondrial GSH depletion and release of soluble intermembrane proteins) can cause a bioenergetic catastrophe culminating in the disruption of plasma membrane integrity (necrosis). 

## 5. Targeting Mitochondria as a Therapy

Some pharmacological interventions have tried to target mitochondria in ALS, but all with very limited success. Interventions targeting mitochondrial dysfunction in ALS have been the subject of a recent systematic review and meta-analysis including 76 studies [127]. This analysis compared different pathway targets, i.e., metabolism, inflammation, apoptosis and oxidative stress, but results rendered no statistical difference in efficacy among these therapies. Nevertheless, animals treated with therapies targeting the mitochondria lived significantly longer than controls, but only if given before the onset of symptomatology [127]. As indicated in the caption of Figure 1, it is suspected that this pathological cascade of events progressivelly damages the MNs until a threshold is reached and cell deterioration and death are irreversible. Therefore, much of this suggestion and the conclusion raised in the systematic review and meta-analysis of Mehta et al. indicate the same finding: the therapy can be more effective if it is administered as soon as possible. In any case, it is essential to discover the origin, the cause that activates this whole cascade. Otherwise, our actions, in the best of cases, can slow down the progression but not prevent it. 

Based on the available evidence, we have selected some compounds which show promising properties. Our criteria to select the following examples rely on the efficacy, at least in preclinical models, to ameliorate the symptomatology and/or extend survival.

Melatonin is an antioxidant and, presumably, decreases oxidative stress [128]. ALS patients taking melatonin had a significantly decreased annualized hazard death rate and a slower rate of decline in the ALSFRS score compared with the non-melatonin users [129]. 

The extracellular signal-regulated kinase (ERK) and phosphatidylinositol 3-kinase (PI3K/Akt) pathways regulate the activation of antioxidant response element-driven antioxidant gene expression. α-Lipoic acid is a natural .OH scavenger and an inducer of the ERK/Akt-dependent pathway [130]. Its potential neuroprotective effect in ALS is now being explored in humans (NCT04518540 Explore Neuroprotective Effect of Lipoic Acid in Amyotrophic Lateral Sclerosis, www.clinicaltrials.gov accessed on 5 June 2021).

The association of nicotinamide riboside and pterostilbene (see above) showed efficacy in ALS patients in a small pilot study (NCT03489200 EH301 for the Treatment of ALS) and is now being tested in a larger trial (NCT04562831 the NO-ALS Study).

Metal chelators, e.g., the iron chelator deferiprone, may also help to limit metal-induced toxicity leading to oxidative stress [131]. A clinical trial showed that the decreases in the ALSFRS scale and the body mass index were significantly smaller for the first 3 months of deferiprone treatment as compared to placebo-treated patients (FAIR-ALS II NCT03293069).

Mitochondria-penetrating lipophilic cations, such as MitoQ and SkQ1, the derivatives of ubiquinone and plastoquinone, respectively, attached to the triphenylphosphonium cation. Both have antioxidant properties (at nM concentrations) and can bind to mitochondrial cardiolipin to prevent its oxidation [132]. MitoQ has shown beneficial effects in animal models, including ALS [133]. 

Pramipexole (PPX), a dopamine analog, reduces oxidative stress [134]. In addition, glutamate-induced dopaminergic neuronal death is blocked by adding PPX to the culture medium, and continuous subcutaneous injection of PPX in rats inhibits the formation of ubiquitinated inclusions in dopaminergic neurons subjected to pro-inflammatory molecules [135]. DexPPX has shown efficacy in ALS patients, reducing the ALSFRS-R decline [136] (NCT01622088). In addition, a human induced pluripotent stem cell-based phenotypic screen of drugs using MNs derived from SALS and FALS cases identified ropinirole, a dopamine D2 receptor agonist, as the top candidate. Interestingly, the beneficial effect of ropinirole was attributed to rescuing mitochondrial dysfunction [137]. 

Reducing abnormal mitochondrial fission may protect MNs. It has been reported that sustained treatment with a peptide inhibitor of the activated fission regulator Drp1, after symptom onset (day 90), significantly reduced muscle atrophy, improved motor function and increased the survival of SOD1^G93A^ mice [22]. Further results suggest that modulation of the protein phosphatase 1-Drp1 cascade may be a therapeutic target in ALS [138].

Additionally, targeting the clearance of damaged mitochondria is a therapeutic strategy that could be implemented. In this regard, some mitophagy inducers have promising benefits to the protection of neurons, e.g., NAD^+^ precursors (such as nicotinamide riboside), urolithin A [139], spermidine [140] or the FDA-approved rapamycin [141] and metformin [142].

Olesoxime (TRO19622), a small cholesterol-like structure, has shown neuroprotective properties for MNs in cell culture and in rodents acting on mitochondria, possibly at the PTP [143]. However, only in the first 12 months of a phase II/III clinical trial did it show an improvement in the motor symptoms of spinal muscular atrophy (MITOTARGET NCT00868166).

Neuroinflammation associates with the release of cytokines which represent potential apoptosis (i.e., TNFα, IFNγ, IL1β) [144], whereas TNFα also interferes with the normal electron flow in the mitochondria and increases the generation of ROS [145]. Therefore, compounds acting against neuroinflammation can also show efficacy. Some interesting options are: a) masitinib, a tyrosine kinase inhibitor, which is predicted to work by decreasing microglia-induced inflammation of MNs in the brain and spinal cord [146] (NCT02588677); b) a combination of sodium phenylbutyrate and tauroursodeoxycholic acid (AMX0035), which may limit cell death and neuroinflammation (NCT03127514, NCT03488524, NCT04516096) [147], and c) cyclic nucleotide phosphodiesterase (PDE) inhibitors to prevent glial cell activation (e.g., ibudilast, NCT02238626 IBU-ALS-1201, NCT04057898 COMBAT-ALS). Interestingly, ibudilast also enhances the autophagy-mediated clearance of ALS-associated TAR DNA-binding SOD1 and TDP-43 aggregates [148].

Despite the fact that the list of drugs under trial or investigation, at different levels, is numerous, it is important to note that none are specific for ALS. They all have, to varying degrees, potential side effects to be aware of. Furthermore, and we believe that this is key, no single drug-based therapeutic approach has had minimally relevant success against ALS to date.

## 6. Conclusions

Genetic testing may identify patients with potential for presymptomatic intervention. However, most ALS cases are sporadic and not caused (as far we know) by a genetic mutation. In addition, there is no known biomarker for the detection of early disease states in SALS. In light of the experimental evidence, oxidative stress and neuroinflammation are the main mechanisms leading to MN degeneration. Damage to mitochondria seems to be a central mechanism in the activation of MN death. Since this damage can be detected early in the disease, targeting patients at or after symptom onset can be critical. There are currently two treatments approved by the U.S. Food and Drug Administration for the treatment of ALS: riluzole (thought to interfere with the activity of glutamate) and edavarone (thought to act as an antioxidant). Both show very limited efficacy. Thus, the urgent need to find new and better therapeutic options remains. Based on the results of many clinical trials run in ALS, a simple conclusion is that single drug approaches have proven ineffective. Based on the complexity of the disease, but also on the accumulated knowledge we already have, it seems coherent to suggest that the use of drug combinations should be our next step. Consequently, a mitochondria-oriented therapy should target multiple mitochondrial signaling and molecular pathways.

## Abbreviations

ALS: amyotrophic lateral sclerosis; MN(s): motor neuron(s); SALS: sporadic ALS; FALS: familial ALS; CNS: central nervous system; TBK1: TANK-binding kinase 1; ROS: reactive oxygen species; RNS: reactive nitrogen species; PrP: prion protein; GSH: glutathione; Nrf2: nuclear factor E2-related factor 2; VCP: valosin-containing protein; Sirt(s): sirtuin(s); PGC-1α: peroxisome proliferator-activated receptor γ co-activator 1 α; HMGCS2: 3-hydroxy-3-methylglutaryl CoA synthase 2; SQSTM1/P62: sequestosome 1; ERK: extracellular signal-regulated kinase; PI3K: phosphatidylinositol 3-kinase; PPX, pramipexole; Drp1: dynamin-related protein 1.
